# Nature-Based Resilience: Experiences of Five Cities from South Asia

**DOI:** 10.3390/ijerph191911846

**Published:** 2022-09-20

**Authors:** Mahua Mukherjee, Deepthi Wickramasinghe, Imon Chowdhooree, Chimi Chimi, Shobha Poudel, Bhogendra Mishra, Zainab Faruqui Ali, Rajib Shaw

**Affiliations:** 1 Department of Architecture and Planning, Indian Institute of Technology, Roorkee 247667, India; 2 Department of Zoology and Environment Sciences, Faculty of Science, University of Colombo, Colombo 00700, Sri Lanka; 3 Postgraduate Programs in Disaster Management (PPDM), BRAC University, Dhaka 1212, Bangladesh; 4 Architecture Department, College of Science and Technology, Royal University of Bhutan, Phuntsholing 13001, Bhutan; 5 Research Department, Policy Research Institute, Kathmandu 44600, Nepal; 6 Department of Architecture, BRAC University, Dhaka 1212, Bangladesh; 7 Graduate School of Media and Governance, Keio University, Fujisawa 252-0882, Japan

**Keywords:** South Asia, cities, urban disaster risk, comparative study, Nature-based Solutions (NbS), Nature-based Resilience (NbR)

## Abstract

As in many other parts of the world, the urban areas of the South Asian region are increasingly expanding. While cities today are the heart of commercial, technological and social development, they are also vulnerable to a variety of natural and anthropogenic threats. The complex urban infrastructure, and the ever-expanding population in cities, exacerbate the impacts of climate change and increase the risk of natural hazards. Throughout history, various hydrological disasters including floods, tidal surges, and droughts, and non-hydrological disasters such as earthquakes, landslides, and storms have led to catastrophic social, economic and environmental impacts in numerous South Asian cities. Disaster risk reduction is therefore central to ensure sustainability in urban areas. Although Nature-based Solutions (NbS) are identified as a promising strategy to reduce risk and increase resilience, there appears to be a lack of evidence-based approaches. NbS are measures that can be practiced to obtain benefits of nature for the environmental and community development through conserving, managing, and restoring ecosystems. Against this backdrop, the South Asian cities provide opportunities to evaluate capacities for achieving Nature-based Resilience (NbR) through NbS. This study documents insights from five cities of five different countries of the South Asian region which are subjected to a wide array of disasters: Barishal (Bangladesh), Phuentsholing (Bhutan), Gurugram (India), Kathmandu (Nepal), and Colombo (Sri Lanka). The primary objective of this study is to provide evidence on how NbS are being practiced. Thus, some success stories in cities under consideration are highlighted: restoration of natural canals through integrated development plans and community participation (Barishal), concepts of Gross National Happiness (GNH) and minimal nature interventions (Phuentsholing), “Greening cities’’ including eco-corridors, vegetation belts, biodiversity parks (Gurugram), proper land use planning aims at different disasters (Kathmandu), and wetland restoration and management with multiple benefits (Colombo). These cases could therefore, act as a “proxy” for learning from each other to prepare for and recover from future disasters while building NbR.

## 1. Introduction

Cities are growing day by day as more global populations prefer urban living. Urban areas contribute largely to, and are influenced by, environmental degradation with changes in land use, depletion of natural elements, and socio-economic activities [1,2,3]. These areas are becoming vulnerable to disasters that are caused either by nature or by humans, resulting in a wide array of socio-economic and environmental impacts [4,5,6].

The South Asian region is home to nearly 25% of the world population, and the urban population has reached more than 1.96 billion. Even though South Asia is not the fastest-growing urban region, a staggering 36.6% of the total population lives in its urban areas [7]. The decadal growth rate of urbanization is captured as very high between 2010 and 2020, with an expansion of 227.8 million in the urban population. The urban living quality is far from ideal in general. The rapid urbanization, coupled with the impacts of climate change and extreme events, makes the region an apt case for exploring the potential for resilience [8].

In the case of achieving resilience, the traditional paradigm of engineering-based policy planning has been shifted to new approaches that consider reinventing nature as an essential component of the ‘urban ecosystem’ [9,10] by allowing nature-based solutions (NbS) [11,12,13].

Approaches to foster and enhance services of nature and ecosystems have gained increased attention in recent decades [14] where new concepts were introduced, including Ecosystem services (ES), Green infrastructure (GI), and Nature-based solutions (NbS) [15].

NbS refers to the use and management of natural ecosystems to enhance the services they provide for the benefit of human beings [16]. NbS entails working with and promoting nature and natural ecosystems to support solving societal challenges [17]. For example, by conserving the forests in an upper catchment, the flood risk of down-stream areas could be reduced [18]. Similarly, reforestation, restoration of wetlands [19], and increased green cover [20] could offer many benefits to urban areas including mitigating impacts of extreme weather events, enhancing cooling effects, recreational potential, etc. [21,22]. On the other hand, human well-being and health could be hampered by the way urbanization and climate change influence ecosystems and the services they provide to society [23,24].

NbSs offer practical solutions for disaster management. In contrast with many engineered solutions, NbSs are equipped to handle the impacts of climate change and offer mitigatory options [25]. By promoting NbSs both climate mitigation and adaptation issues could be solved at a relatively low cost [26]. On the other hand, Ecosystem-based Disaster Risk Reduction (Eco-DRR) is supplementary to NbS. Eco-DRR highlights the necessity of ecosystem conservation, restoration, and sustainable management to receive nature’s “free services” to reduce disaster risk [27].

The positive link between NbSs and urban disaster resilience is endorsed by contemporary researchers [28]. NbSs have gained the attention of the city planners and policymakers who struggle to manage urban areas where a variety of issues exist. Moreover, NbSs are currently reflected in the strategic goals of many urban plans across the world as part of city rehabilitation efforts to re-brand, re-vision, and re-orient themselves to be more friendly, livable, and attractive to tourists and visitors [29]. Conversely, nature’s free services offer a transformative social influence because they mediate new social relationships and configurations that foster social innovation in cities [30] and influence how people see nature and their relationships with it in urban settings [31].

Against the backdrop of rapid urbanization where poverty and population expansion among many others, add a compounding effect, cost effective solutions could be attractive to solve issues in cities. In this context, the prime focus of the present article is that learning and sharing experience from similar cases in the region. It is essential to managing uncertainties in the urban environment. Yet, there are no evidence-based multi-country or multi-city studies from the region to explore the need and opportunity for enhancing nature-based resilience, and the paper attempts to fill this gap.

Accordingly, this study aims to investigate five selected cities across five countries in the South Asian region to obtain a region-specific representation of the issue. The principal research question raised in this study is: What are the different NbSs practiced in each city to reduce disaster risk and increase resilience?

Particularly, the current study attempts to present urban risk, preparedness, and capacity for NbS in each case. The results of this endeavor could present important insights for the cities in the region with a similar environmental and socio-political background to adopt the good practices reported. Moreover, the current research will provide some “impetus” for the other cities to learn from and take action.

## 2. Study Framework and Methodology

All selected cities have been experiencing one or many natural disasters, either directly or indirectly related to changing weather patterns (e.g., floods and drought). Some cities, on the other hand, are prone to geological hazards such as earthquakes, and landslides. However, in all cases, impacts of such disasters have been significant. In this study, first the research team explored that all cities could be comparable in relation to disasters and their management with a flavor of natural infrastructure. Development of profiles on five selected cities from five countries was the very first step of the study: Barishal (Bangladesh), Phuentsholing (Bhutan), Gurugram (India), Kathmandu (Nepal), and Colombo (Sri Lanka) in the South Asian region. Parameters used for selecting cities include a diversity of natural disasters that happened, risk-proneness, and prevailing endorsement of NbR either through policy or action plans or both.

The co-authors of each country studied the representative cases. A mixed methods approach was employed to collect contemporary geographic, socio-economic, and development information in the cities. A special emphasis was given to the contemporary NbSs practiced to mitigate impacts of disasters. A structured study framework was developed across selected cities to assess the NbR status. Accordingly, city briefs including risk profile, assessment, and resilience measures in place, were documented.

## 3. Selected City Case studies

### 3.1. Case 01: Barishal, Bangladesh

#### 3.1.1. City Profile

**Figure 1:**
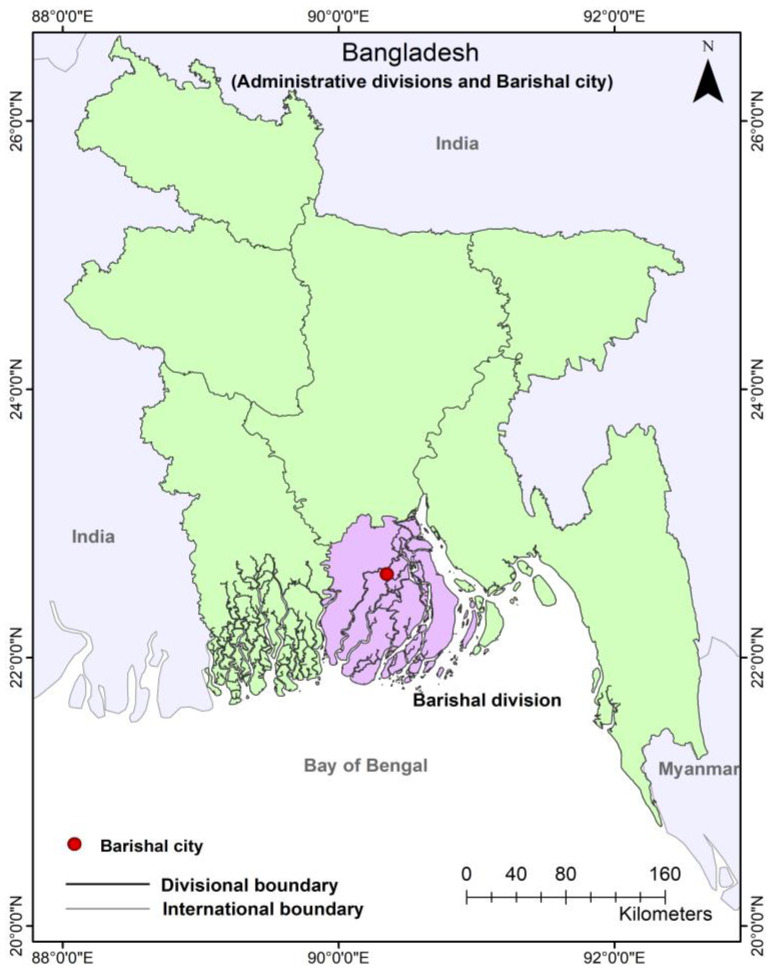
Map showing the location of Barishal city in Bangladesh (Authors, 2022).

Barishal is a major river-port city (Figure 1) that lies on the west bank of the River Kirtankhola and within 106 km of the Bay of Bengal in the south-central coastal region of Bangladesh [32]. It is also the divisional headquarter of the Barishal division. Almost 0.5 million people are living in its City Corporation area (58 km^2^) which lies between 22°30′ to 22°45′ north latitude and between 90°18′ to 90°23′ east latitude [33]. The city is crisscrossed by multiple rivers and almost 24 natural canals [34], and on average, its elevation is only 2.5 m higher than the sea level [33].

#### 3.1.2. Risk Profile

The urban environment is highly challenged due to unplanned development, drainage congestion, lack of proper solid waste management, and encroachment of rivers, canals, and other water bodies [32]. The city experiences cyclones and flooding (waterlogging, riverine/monsoon flood, and storm/tidal surge) almost every year as its significant natural hazards where anthropogenic causes exacerbate the condition [35,36]. The city has experienced assets and infrastructure damages due to catastrophic cyclones in 1991, 2007, 2009, 2013, 2016, 2020, and 2021 and floods in 1988, 1991, 2004, 2007, 2010, and 2020 in recent times. Almost every year, monsoon floods and cyclones cause damage of USD 10 million, which will significantly increase by 2050 [32]. Other hazards include tornados, salinity intrusion, riverbank erosion, heavy rainfall, extreme temperature, drowning, fire accident, industrial hazards and pollution, road and naval accidents, arsenic contamination, etc.

The city is partly served with an open drainage system and largely depends on natural drainage through existing canals. However, the natural water flow of these canals is hampered by encroachment and illegal settlements [37]. The situation has been exacerbated because of siltation, the dumping of solid waste, and discharging sewerage into the canals [34].

#### 3.1.3. Risk Assessment

Risks of this city were assessed at various times, mainly for preparing the development planning proposals. One significant work is the Vulnerability and Feasibility Assessment conducted in 2015 which was funded by KfW Development Bank [33]. A Facebook group is operated, titled “Barisal—Problem, and Prospect”, to obtain feedback and suggestions from city dwellers, especially the younger generation [38].

#### 3.1.4. Resilience Measures

Planning documents including the Structure Plan 2010–2030, Urban Area Plan 2020–2030, and Detail Area Plan highlight the necessity of reviving rivers and canals to achieve nature-based resilience [39]. Several projects and programs are planned and implemented to restore natural canals to facilitate natural drainage to reduce waterlogging. These canals serve multiple purposes: naval transportation [34], freshwater sources, stormwater drainage, areas for aquaculture and biodiversity, irrigation, and means to create a comfortable microclimate [39].

In 2015, Sagardi canal was developed as a as a demonstration project that intended to reclaim encroached lands, remove sewerage inputs, and restore. The periphery was developed to include pedestrian pathways, bicycle lanes, and urban greenery. New bridges and culverts were introduced for smooth naval transportation [34]. The project acts as a nature, infrastructure and community hub.

In 2016 another project was launched as the Climate Change Adaptation for Urban Areas (CCAUA) Programme, which aimed to make the city climatically resilient and environmentally sustainable. This transformative urban restoration project aimed at reducing pollution including sewage and solid wastes, enhancing infrastructure, increase disaster resilience and overall improvement of health and well-being of people [33].

#### 3.1.5. Nature-Based Resilience Assessment

**Figure 2:**
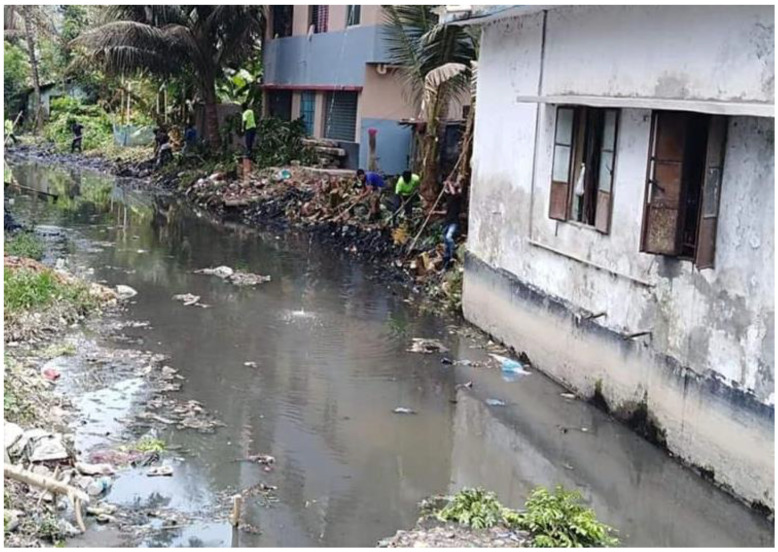
Volunteers cleaning up the Jail canal in Barishal in 2016 [38].

Jail canal is a significant canal, the condition of which has been investigated through reviewing newspaper articles [40,41,42] and interviewing key informants. This 3.2 km long canal, which is connected to River Kirtonkhola, was restored in 2016 by Barishal District Administration, and Barishal City Corporation. More than 4000 local people worked voluntarily (Figure 2) which is an example for successful community participation. Following removal of illegal settlements, blockages, solid waste dumping, and sewerage inputs, the canal regained a smooth flow. The success story influenced the people of other areas. Thus, citizen-driven initiatives were taken to revive other canals too.

Unfortunately, at the end of 2019, the Jail Canal and other canals had lost their flow, due to resettlements of people who left in 2016. Among many reasons, the significant causes of degradation of canals include lack of proper development guidelines and provisions to restrict unsustainable activities, poor awareness among local residents about the project, and little attention paid to integrated planning and implementation.

This project highlights the need of integration of project components to protect canals and bank areas, make the canals navigable, and ensure the water quality that may allow ecosystems to function. Awareness raising and encouragement to continuous community participation are essential. These initiatives as means of NbSs are also needed to be successful to avoid future impacts of climate change.

### 3.2. Case 02: Phuentsholing, Bhutan

#### 3.2.1. City Profile

**Figure 3:**
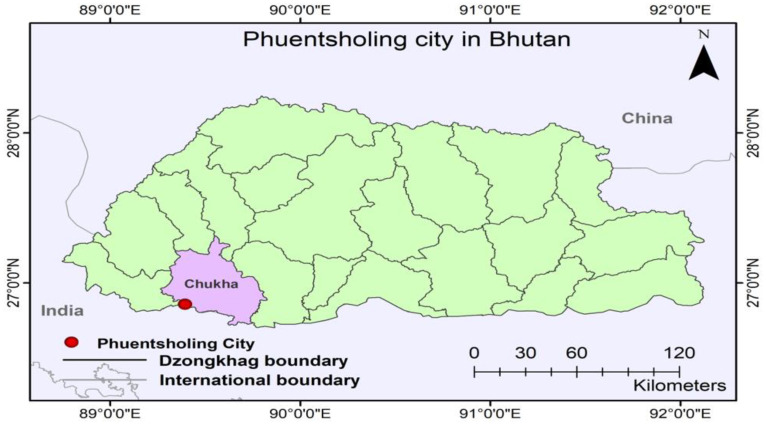
Map of Phuentsholing City in Bhutan (Authors, 2022).

Phuentsholing (Figure 3), the second-largest city in Bhutan, after Thimphu, is perched on the Himalayan foothills adjacent to the Indian border town *Jaigon* of West Bengal, covering an area of 19.68 km^2^ [43]. It is located at 26°49′ to 26°54′ north latitude and 89°20′ to 89°28′ east longitude with an elevation of 160 m above sea level [44]. Due to its location on the Indo–Bhutan border, it has served as the leading center for its economic activities. It is known as the financial gateway to Bhutan. Phuentsholing has a population of 27,658 against the total population of Bhutan of 727,145, according to [45]. Phuentsholing is located in the sub-tropical climate zone, having hot and humid summers with a maximum of 32.5 °C (June), and warm and dry winters with a minimum temperature of 13.3 °C in January [46]. The city receives an annual rainfall of around 4000 mm, with a maximum occurring from June to September [43].

#### 3.2.2. Risk Profile

**Figure 4:**
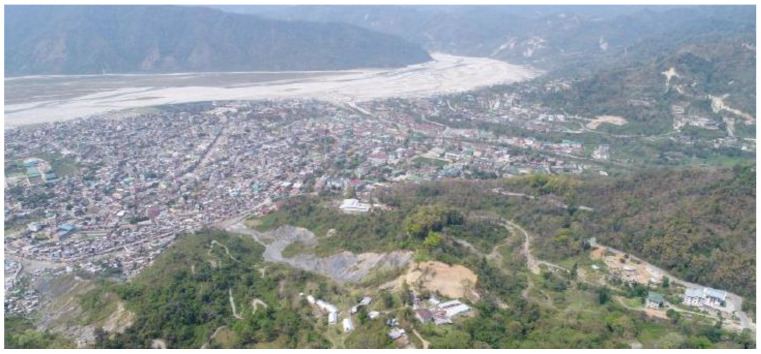
Arial view of Phuentsholing city, Bhutan [48].

**Figure 5:**
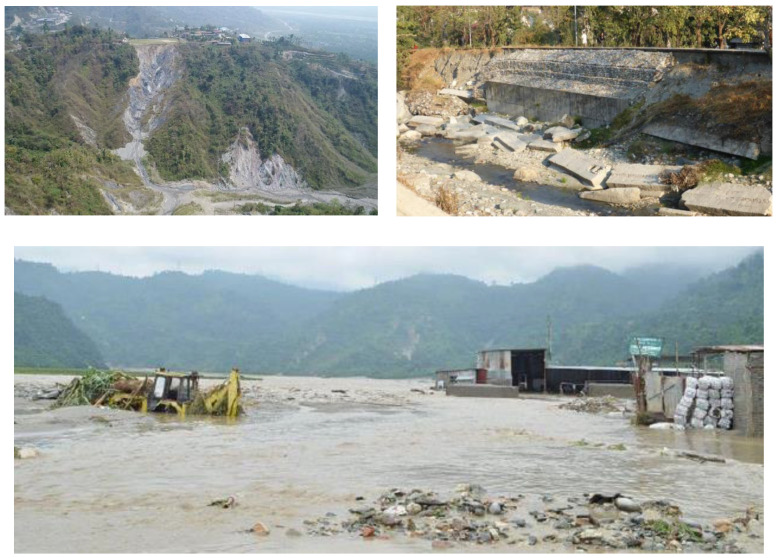
Disasters in Phuentsholing [48,49]. (**top left**—Landslide damage, **top right**—flood damage; **bottom**—Devastating floods in the city).

Phuentsholing (Figure 4 and Figure 5) is highly vulnerable to annual flash floods as it is situated on the bank of the *Amochu* River and *Omchu* stream. Significant floods of 1993, 1996, 2000, 2009, 2015, and 2016 caused many impacts, including disruptions of community lives and commercial activities [47]. Its location on the boundary between the Indian and the Eurasian tectonic plates has made it vulnerable to earthquakes. As it falls under Zone-IV and V of the Indian seismic zonation map it is expected to experience earthquakes of magnitude ranging from 5.8 to 6.6 Richter scale [47]. The city is also vulnerable to landslides, windstorms, and mosquito-borne diseases such as dengue and malaria. Air pollution from the *Pasakha* Industrial Area is another risk [43]. Moreover, the drug and tobacco smuggling business along the porous Indo–Bhutanese border also poses risks to the city’s safety.

With limited land for urban infrastructure development, its urban area has been experiencing continuous pressure due to rapid urbanization.

#### 3.2.3. Risk Assessment

The Royal Government of Bhutan (RGoB) and external agencies have assessed the risks several times. Moreover, the Phuentsholing *Thromde* (municipality) has also evaluated its six constituencies as documented in the Thromde Disaster Management and Contingency Plan 2018 [43]. However, the National Adaptation Programme of Action (NAPA II) project on climate change (2014–2018) and Disaster and Climate Risk and Vulnerability Assessment for Phuentsholing Township Development Project (2018) were the two significant landmarks. The Global Environment Facility (GEF) funded the NAPA-II to enhance resilience to climate change impacts and natural disasters [48]. These documents have also identified possible landslides and flood disasters. Similarly, the Asian Development Bank supported the Disaster and Climate Risk and Vulnerability Assessment, 2018, focused on water-induced disasters, particularly floods [50].

#### 3.2.4. Resilience Measures

As the primary document for controlling urban development, the Phuentsholing Structure Plan (PSP) 2013–2028 has segregated different land uses based on topography and geological conditions from an economic perspective. Vulnerable areas were identified including environment conservation precincts, where construction is not allowed and Agri-based environment, where only two-storied buildings are permitted. Similarly, the National Environmental Protection Act of Bhutan 2007 and the Water Act of Bhutan 2011 provide legislation on developmental activities close to water resources. The 11th five-year plan provides funds to enhance safety, promote community vitality, and make Phuentsholing a highly livable town by constructing bicycle ways and footpaths, recreational parks, and sports facilities. It has also included measures for disaster management and risk reduction, highlighting hazards such as landslides and flood [51]. Evaluation of the success of these projects are carried out by Annual Performance Agreement and the compliance report prepared by the Department of Human Settlement (DHS) [52]. The DHS reviews the compliance and development activities, and recommends necessary actions [49]. The land-use planning and maintaining buffers and setbacks according to acts and regulations ensure the expansion of urban green with proper walkways. For example, the compliance report 2016 shows that the developed green area in zone 1 is 7% against 5% as per PSP 2013–2028 [49]. The agreement and periodic reviews promote the concept of nature-based resilience through practicing Bhutan’s philosophy of middle way development as per the four pillars of Gross National Happiness.

#### 3.2.5. Nature-Based Resilience Assessment

The authorities have planned to develop selected riparian land in Phuentsholing along both sides of the *Amochhu* River under a long-term *Amochhu* Land Development and Township Project (ALDTP). This ALDTP project aims to develop smart urban infrastructures to support residents with proper measures to mitigate flood and erosion risks and enhance safety. The lessons learnt will be used in efficient city development integrating with existing urban fabrics and preserving Bhutanese architectural heritage. Along with constructing a 15 km long riverbank protection wall, it has incorporated NbSs including slope stabilization, bioengineering techniques, soil nailing, surface protection using geosynthetics and membranes, and soil retention methods using gravity walls, etc. Moreover, in addition to structural measures for flood protection such as embankments and hydraulic structures, the project also has incorporated non-structural measures, including flood forecasting, early warning, raising public awareness on flood preparedness, arranging necessary reliefs, and launching insurance schemes [50,53].

Through reviewing project documents and consulting with key informants, the project adopts the “top-down” process for imposing climate change models and the “bottom-up” approach by assessing community vulnerabilities and promoting community-based flood management procedures. NBR has been achieved by practicing the middle way development philosophy of Gross National Happiness that has played a vital role in disaster risk reduction through minimal interventions into nature. (Risk Profile and Resilience Assessment is somewhat contradictory).

### 3.3. Case 03: Gurugram, India

#### 3.3.1. City Profile

**Figure 6:**
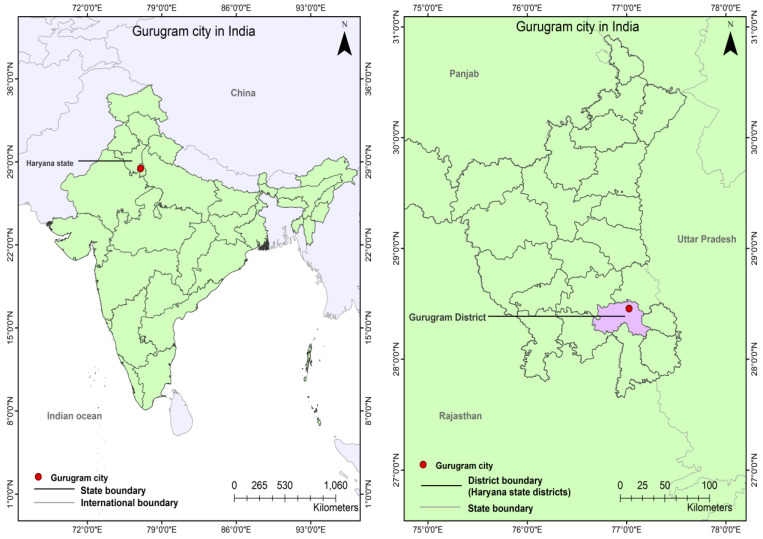
Map showing location of Gurugram city in India (Authors, 2022).

Gurugram city (Figure 6) (also known as ‘Gurgaon’ in Gurugram district, Haryana, India is located at 28.4595° N and 77.0266° E. It has a “Bsh” i.e., Mid-Latitude Steppe and Desert Climate. The population of the Gurugram Municipal Corporation (GMC) area (250 km^2^) [54] has increased from 876,969 (2011) to 999,745 in 2020 with a density of 4000/km^2^ [55]. The Gurugram Metropolitan Development Authority (GMDA) Master Plan covers a 338 km^2^ area [56].

The city’s economy depends on industrial production, and corporate and IT Industries. Aravali hills, the River Yamuna flood plain, Najafgarh canal and jheel (lake), and Ghata lake are the major natural elements that have predominantly characterized the ecosystems of Gurugram for the last four decades.

#### 3.3.2. Risk Profile

Gurugram city faces threats from multiple types of hazards: earthquake, flood, drought, hailstorm, environmental degradation, heat waves, smog, air pollution, lightning, fire, industrial hazards, epidemic, accidents etc. It experiences regular urban floods during the monsoon season and, especially since 2014, severe air pollution during winter, and heat concentration in summer. Severe droughts were observed in 1987 and 2001. Since 1978, the city has been regularly inundated due to the overflow of some canals (i.e., Barsati Nallahs, Hills Torrent). It is in seismic zone IV, and 8.0 is the maximum expected earthquake magnitude [54,56].

Despite its vibrant economy, the socio-economic pattern that aggravates the vulnerability includes an influx of young floating migrants, the vast disparity between income and housing, and the high cost of living. Shrinking agriculture/horticulture land area, lack of critical infrastructures (such as public transport, drinking water, etc.), and impacts of criminal activities on the lower-income group are other issues of concern for this city and livestock [57]. The urban poor have become the main vulnerable group for disasters. The existing drainage system is overburdened, and the natural drainage system is affected by large-scale real estate development.

#### 3.3.3. Risk Assessment

**Figure 7:**
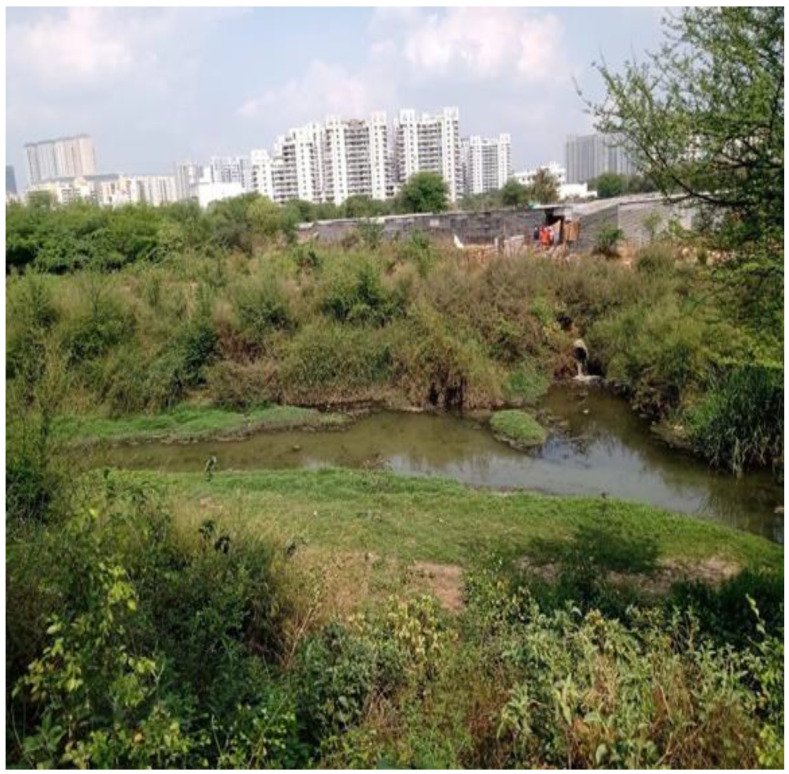
Fragmented blue-green elements in Gurugram (Field Survey, 2021).

Rapid urbanization, loss of blue-green infrastructures, recurrence of urban flooding, water crisis, heat-island coupled with a heatwave, and air pollution affect the city and physical and mental well-being of inhabitants (Figure 7).

Recently, the government introduced an innovative solution to make all stakeholders connected through launching the ‘One Map Gurugram’ [58] service and the ‘myGurugram’ mobile app (Google App store) to understand the risks better. ‘One Map Gurugram’ is a centralized GIS integrated Decision Support System with spatial information for citizen engagement with GMDA and district authority. Multi-hazard zonation and vulnerability analysis can be added to it. The ‘myGurugram’ mobile app is aimed at multiple services to connect citizens and administration, including disaster events and complaints reporting.

#### 3.3.4. Resilience Measures

Gurugram has introduced the Livability Index and is gradually improving its performance in the ‘Ease of Living Index and the ‘Municipal Performance Index’ [59], yet, resilience planning and action are limited.

#### 3.3.5. Nature-Based Resilience Assessments

The city has introduced some NbSs including green belts along roads, biodiversity parks, and eco-corridor to enhance “natural elements” in the city and address urban environmental risks. Ten ‘Miyawaki Urban Forests’ have been planned to develop as urban forests to increase green cover. This method attracted criticism as an expensive short-term solution for long-term environmental problems [60]. The Miyawaki method helps to grow urban forestry quickly in a limited area. The technique is gaining acceptance in Indian cities, yet ecologists and landscape professionals have reservations about disrupting natural ecology and its high cost. Gurugram belongs to the dry-deciduous forest ecosystem; any quick remedial implementation raises such doubt. The city produces approximately 435 MLD (Minimum Liquid Discharge) of wastewater; reuse of treated wastewater has started within city limits [60]. The district administration introduced eight nature-based ‘root bed zone’ and ‘decentralized wastewater treatment (DEWAT). The wastewater treatment plants of 100 to 500 KLD (Kilo Liters per Day) capacities are outside the city as they need larger land parcels [60].

The authority publishes reconnaissance reports based on field data and images of all extreme events. The impact of these reports is yet to be felt, but a dialogue with different stakeholders such as the community, professionals, and NGOs have taken place. The GMDA is interested in applying advanced tools and techniques, such as the Geospatial Mapping of Natural Ecosystem (GeoSM-Nate) Framework and engaging professional consultants to develop neighborhood to city level resilience [59].

Citizens’ psycho-physical wellbeing is positively impacted as the above-mentioned blue-green elements provide areas to relax, exercise, and have social interactions; to reduce noise and air pollution; protecting local biodiversity and greenery within the city.

The GMDA, creates and implements the nature-based Blue-Green Infrastructure (BGI) network. A comprehensive area-level project on developing solutions for Wazirabad Lake for performance can be cited as one example. Through this project, rejuvenation of existing lakes using rainwater and treated wastewater (hybrid NbS and engineered solution), bioswale, and vegetation are planned. Community participation is another pertinent aspect of the same. The GeoSM-NatE Framework was applied for the risk and intervention potential identification stage [59].

For Gurugram, NbS is a feasible critical intervention option to strengthen urban resilience. Contextual integration with engineered infrastructure as a hybrid solution for risk reduction is explored with the GeoSM-NatE Framework application. Accordingly, a few potential blue-green elements are identified to enhance opportunities for BGI networking:

Urban Green: enhanced vegetation cover helps mitigating climate change impacts, reduce disaster risks, and provides a variety of other ecosystem services that support community well-being.

Urban Forest: Traditional integrated agro-forest management with dry-deciduous plants enhance green cover with other positive socio-economic impacts. Herbs, shrubs, and small and large trees add medicinal, commercial, supporting biodiversity, shading, and aesthetic purposes. New options such as Miyawaki are essential to look at but not at the cost of long-term ecological imbalance.

Bioswale and green boundary: Road-side existing green-strip acts as bioswale; little depth will increase retention volume while traveling, scope to sponge, attest pollutants, and attenuate peak discharge to storm drainage network.

Retention/detention Pond: this system helps managing storm and excess flood-water. Rejuvenation of existing detention ponds and lakes will increase the city’s water resilience.

Wastewater treatment and reuse: NbS-integrated hybrid solution for wastewater treatment is cost-effective and involves low-skill maintenance. Innovative use patterns can create alternative water-source and attractive urban public spaces.

### 3.4. Case 04: Kathmandu, Nepal

#### 3.4.1. City Profile

Nepal is one of the fastest urbanizing countries in South Asia [61]. Most of the urban growth is concentrated only in the capital of Kathmandu; the decadal population growth rate of Kathmandu is 61.23% [62]. The population and density of Kathmandu valley are 2,517,023 and 4416 people per square kilometer, respectively, as per the 2011 census [62]. Its latitude and longitude are 27.7172° N, and 85.3240° E. The city belongs to the “Cfa-Humid subtropical climate” of the Koppen classification code. It is situated in the central hills zone at an average elevation of 1350 m above sea level. The city is the hub of the economy as well as cultural heritage. The surrounding mountains and rivers (Bagmati and Bishnumati) are major natural elements that shape its ecosystems. Deforestation in the surrounding hills, riverine pollution, unplanned urbanization, and diminishing agricultural land and open spaces make the valley a high-risk multi-hazard zone, particularly earthquakes, fires, floods, land subsidence, pollution, and landslides.

#### 3.4.2. Risk Profile

**Figure 8:**
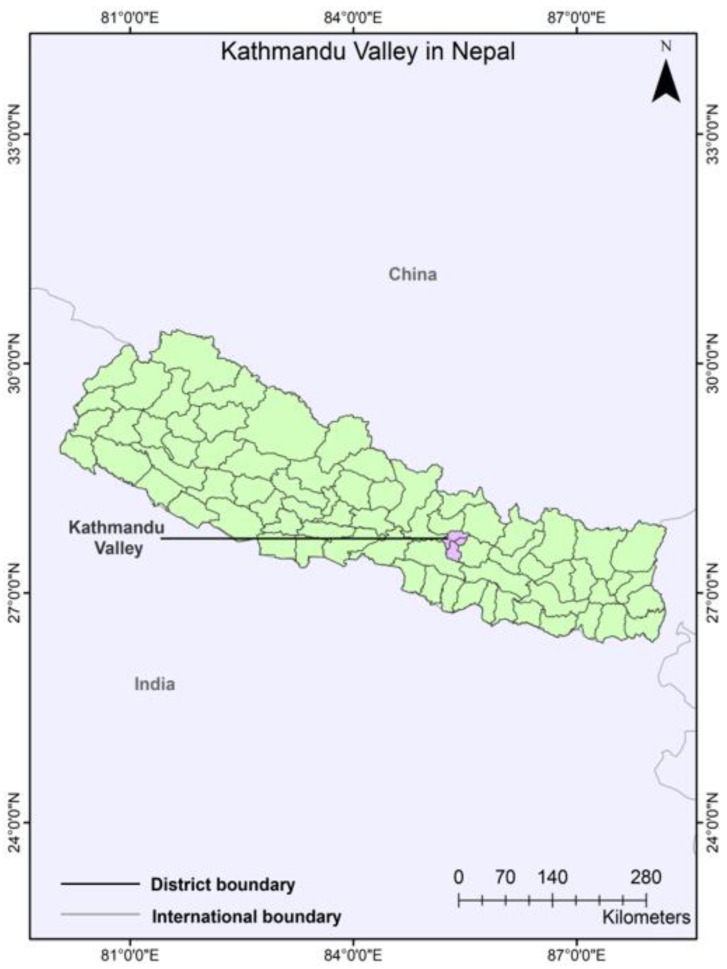
Map showing Kathmandu Valley in Nepal (Authors, 2022).

Kathmandu is at risk of natural hazards and human-induced disasters such as earthquakes, floods, landslides, accidents, air pollution, epidemic, and fire [63]. Kathmandu has experienced major earthquakes and other natural disasters frequently. Widespread occurrence of earthquakes of Richter scale magnitude of 5–6 is expected, a few of magnitude 6–7, and occasional incidents of magnitude 7.0–8.5 shocks occurred in the years 1833, 1934, 1936, 1954, 1965, 1966, 1980, 1988, 2011, and 2015 [3,64]. There was a catastrophic flood in Kathmandu in 1993. Similarly, there are urban floods and inundation incidents almost every year in the rainy season. The overall risk profile of Kathmandu is shown in Figure 8.

#### 3.4.3. Risk Assessment

Kathmandu is facing increasing environmental challenges that threaten the resilience of the city. Unplanned urban development, increased population, poverty, poor housing conditions, unregulated encroachment of rivers, excess extraction of groundwater, and extinction of open spaces lead to exposure and consequently vulnerability to different types of hazards [63]. Furthermore, climate change intensifies disaster risks [65], especially flooding and landslides triggered by heavy rainfall during the monsoon season [66]. The scarcity of open lands and conversion of fertile agricultural land into built-up areas have led to more severe annual flooding. In the past years, the Kathmandu valley has lost most of its forest area, resulting in soil erosion, landslides, and siltation in and around the catchment area. Rivers and streams are primarily used as dumping sites for all types of waste. Urban air pollution in Kathmandu is one of the worst globally, negatively impacting humans, animals, and vegetation [61]. Poorly constructed buildings of informal settlements, combined with limited access to essential services, increase exposure to natural and human-induced disasters.

#### 3.4.4. Resilience Measures

##### Kathmandu Urban Development Project

This project was initiated in 1993 to improve productivity and the urban environment in Kathmandu through physical works, institutional, and policy development, emphasizing local resource mobilization. The main objective was to carry out environmental improvements and increase local resource mobilization to ensure the sustainability of investments. Municipal Infrastructure Improvements included:

(1) upgrading land in the Kathmandu core area, surface, drainage improvements and solid waste management improvements
(2) stormwater drainage improvements over 16 km to cover several catchment areas throughout the city
(3) environmental improvements in the Bishnumati corridor by removing debris, landscaping, and planting, providing three ramps for construction waste disposal.

Bishnumati Link Road (BLR): A 2.8 km road with a 20-m right-of-way and an associated bridge over the Bishnumati River with plantation along the riverbank. It helps to ease access to the road during an emergency. Plantation in the riverbank enhances the natural beauty and ecosystem of the surroundings.

#### 3.4.5. Nature-Based Resilience

##### Kathmandu Metropolitan City Risk Sensitive Land Use Plan

Kathmandu Metropolitan City Risk Sensitive Land Use Plan is based on an integrated sectoral approach and cooperation between urban, national, and international actors [67]. The Plan identifies the available environmental resources as crucial for achieving safer environments. It focuses on flood-prone areas, cropland critical for food production and supply, and urban parks and open spaces due to their importance for recreation and providing shelter during emergencies. At the same time, it shows the potential for mainstreaming ecosystem-based solutions, for instance, through improved land-use planning, reforestation, slope stabilization, and conservation of flood retention areas, into an improved urban governance scheme [68].

##### High Powered Committee for Integrated Development of the Bagmati Civilization

The main objective of this High-powered Committee is to keep the Bagmati River and its tributaries clean by preventing the direct discharge of solid and liquid wastes into the river and to conserve the river system within Kathmandu. To achieve these objectives, the following activities are mandatory to this committee, i.e., construction of the trunk sewer pipeline along both the banks of the river, wastewater treatment plants, roads, and green belts along the banks of the river.

Urbanization has changed Kathmandu into a densely populated and built-up urban landscape. Nature-based resilience is needed to achieve to integrate a range of ecosystem-based approaches to address unprecedented range of challenges including urbanization, disasters, and climate change.

##### The Potential of Nature-Based Resilience of Kathmandu Include

Proper urban planning with open spaces to help reduce disaster impacts by maintaining areas for shelter during an emergency and groundwater recharge and supply.

Maintaining vegetation cover on hillsides can stabilize steep slopes. It has the potential to reduce impacts of earthquakes as well as rainfall-induced landslides.

Integration of Blue, Grey, and Green Infrastructure which play an imperative role in urban disaster risk reduction and management. Grey infrastructures such as dikes, and drainage systems are for stormwater management, while green infrastructures are for parks, and tree-lined streets, and blue infrastructures include ponds, wetlands, rivers, and lakes.

Because of its diverse range of vulnerabilities, nature-based resilience has a high potential to revive the city and enhance its resilience. Nature-based resilience such as maintenance or recovery of riverine and open space encroachment, reforestation of hills for stabilization, banning garbage disposal in the river, and the selective creation and maintenance of urban green areas for recreation will reduce disaster and climate change-related risks in the valley to a considerable degree.

### 3.5. Case 05: Colombo, Sri Lanka

#### 3.5.1. City Profile

**Figure 9:**
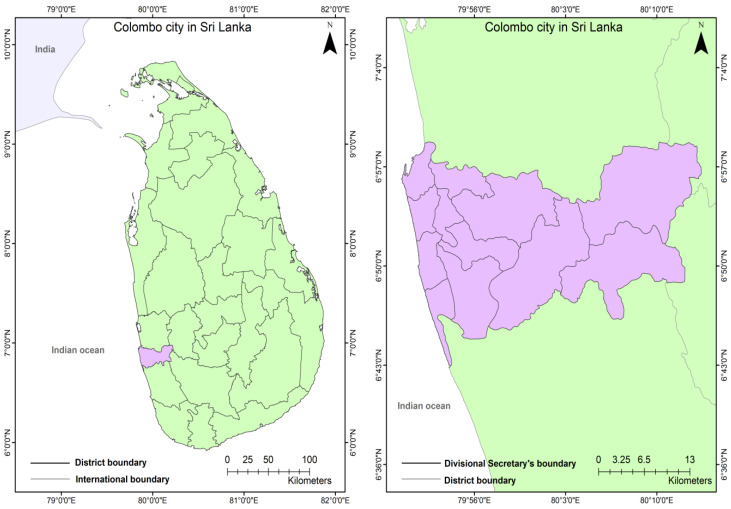
Map showing the location of Colombo city in Sri Lanka (**left**), and its City Corporation area (**right**).

Colombo (Figure 9) is a coastal city with relatively flat terrain, ranging from two meters below mean sea level (MSL) to 18 m above MSL. The city limits cover an extent of 14.41 km^2^ and are situated in the 6.9271° N, 79.8612° E coordinates. With 14 administrative sectors, Colombo is among the most densely populated areas in the country, contributing to 40% of the GDP and covering significant socio-economic aspects. Colombo is the central commercial city and the control center of the country’s banking and financial organizations. The population in 2001 was 642,000, and the people experienced an average annual increase of less than 1%. The extent of the ‘floating’ population is estimated to be approximately 500,000 currently.

#### 3.5.2. Risk Profile

Colombo has been highly vulnerable to flash floods for the past three decades. The city is prone to floods because of its location in a low-lying river basin close to the Indian Ocean [69]. The Southwest monsoon that hits in May–June brings extra rain, contributing to flooding and inundation of urban areas. In 2010, 2016, and 2017 the city experienced extreme events that resulted in the inundation of roads and lands, disruption to community lives and commercial activities, health impacts, and even deaths [70].

Among other hazards, the 2004 Indian Ocean Tsunami hit was a significant event where hundreds of people on the coast were affected, yet, Colombo had a relatively low impact compared to the other coastal areas. Effects of tropical cyclones have been felt but are not very frequent. Colombo is vulnerable to lightning hazards due to climatological changes. An increased number of days with high temperatures because of changing climate has been observed recently. More than 1000 flood events were evident between 1974 and 2008 because of hydro-meteorological hazards initiated by the changing climate [71].

#### 3.5.3. Risk Assessment

Developmental and population pressure have resulted in the filling of lowlands, and these encroachments have made the city and its people vulnerable to floods. The Kelani River, which is among the country’s key rivers, flows to the Indian Ocean while going via the city [72]. Even though the river flooding has been managed with a barrier, most low-lying areas along the river basin are vulnerable to floods [73,74].

Urban vegetation cover has been decreasing in the city at an alarming rate. Increased infrastructure, including buildings, roads, car parks, and impervious surfaces, presents the risk of water accumulation for many hours and even days. A decades-old, outdated stormwater canal network system that is insufficient to handle water surges aggravates the situation. Inadequate policy support and implementation, poor coordination, incompatible modes of operation among agencies, weak enforcement of laws, and negligence in monitoring have created a significant degree of discontent in urban flood management.

#### 3.5.4. Resilience Measures

Resilient cities can absorb many stresses and lessen their impacts making urban areas livable and healthy [75]. Loss of vegetation, alteration of natural habitats, and wetlands have made Colombo more vulnerable to disasters, including extreme weather events and impacts of climate change [76]. The government of Sri Lanka has taken a central role with the involvement of multi-stakeholders for effective urban improvement, especially to achieve targets of the Sustainable Development Goal (SDG)s and the Paris Agreement. Together with government agencies, various other institutes engage in increasing green cover, restoration, and conservation of the natural environment, which is the sine qua non or essential requirement for urban resilience.

#### 3.5.5. Nature-Based Resilience Assessments

This study examines the Metro-Colombo Urban Development (MCUD) project that emphasizes developing wetlands as vital parts of nature’s wealth. Even though often neglected and underestimated, wetlands provide services worth trillions of US dollars annually for free [77]. These ecosystems are among the world’s most productive and beneficial landscapes [78]. They help clean the atmosphere by absorbing carbon dioxide and releasing oxygen (the ‘lung’ function), filtering pollutants (the ‘kidney’ function), influencing the hydrological cycle, and reducing the risk of extreme events such as floods and droughts [79]. These areas can be identified as ‘biological supermarkets’ that accommodate biodiversity home to many plants, animals, and microbes.

In 2012, the MCUD Project increased urban resilience and reduced flood impacts in the Metro Colombo drainage basin, 105 km^2^ in size. The project encompasses improving the natural flood reduction network, including canals, lakes, and wetlands in Colombo and its suburbs. The Sri Lanka Land Development Corporation (SLLDC) and the Colombo Municipal Council (CMC), as the most prominent local authorities in the country, are the key stakeholders of this project [80]. The project is supported by a variety of government and local government institutes [81].

**Figure 10:**
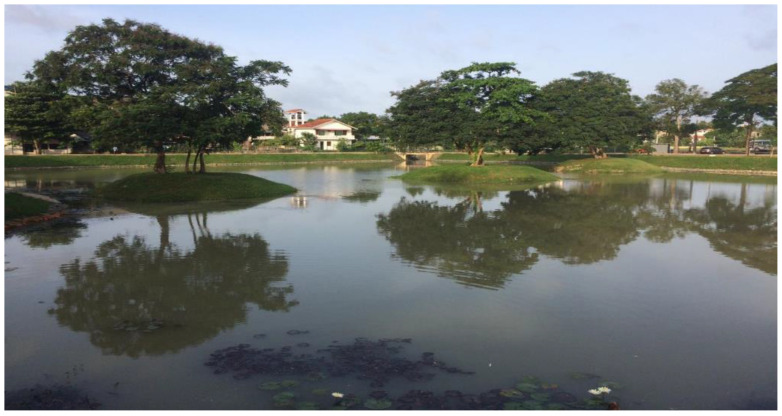
A restored wetland as a part of city-wide wetland network (Field survey, 2021).

**Figure 11:**
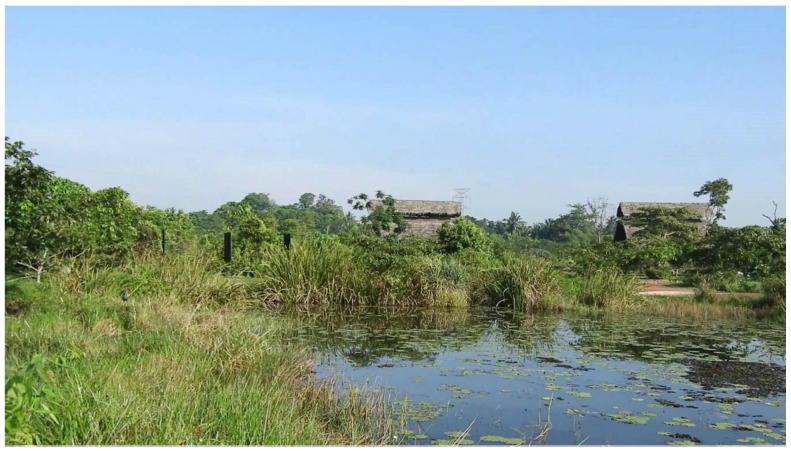
Diyasaru water park—a newly renovated urban wetland with multiple functions including recreation and research opportunities etc. (Field survey, 2021).

One of the main and unique components of this project is the creation and maintenance of wetland parks (Figure 10 and Figure 11). The Ramsar Secretariat endorsed Colombo as one of the world’s eleven wetland cities in 2018, owing to its significance to accommodate these aquatic habitats of multiple uses [82]. Creating wetland parks closer to nature, acting as green and blue infrastructure to mitigate disaster impacts, and increasing resilience indicates that Colombo is going greener. The parks serve as places of education, recreation, research, and leisure activities while generating income for the government as well as mitigating urban floods. The estimated recreational potential of these parks is around USD 13 million a year, and the Colombo Metropolitan Region may lose 1% of its GDP on average annually if it is flooded [80]. The following two parks, which are two vital parts of the network, serve as improved ‘blue-green’ natural infrastructures with multiple benefits.

#### 3.5.6. Beddagana Wetland Park

Situated in the suburbs of Colombo, this park (32 ha) provides recreational and educational opportunities for adults, youth, and school children. The wetland serves as a haven for urban biodiversity, supporting many species, including colorful butterflies, birds, and small mammals. During the migratory season, many winter visitors add to the bird fauna in the wetland [83].

#### 3.5.7. Diyasaru Uyana (“A Park with Plenty of Water” in the Local Language)

Situated in a low-lying area near the parliamentary complex in Madiwala, this urban wetland park of 60 acres is considered a paradise for many animals and plants. It accommodates rare fishing cats, otters, reptiles, mammals, and many species of birds with a dedicated zone for butterflies.

In this park, isolated patches of wetlands are connected through a network of canals which make the area act as a huge wetland habitat. The wetland provides educational and recreational opportunities, including an environment-friendly children’s park, night camp facility, study center, bird watching tour, butterfly garden, educational tours, boat rides, leisure activities, etc.

The two wetland parks contribute to livability of cities and help providing, regulating, cultural and supporting ecosystem services with many climate change co-benefits. Additionally, these NbSs make the city more resilient as the nexus of water, land, and biodiversity help enhance urban communities’ adaptation capacities, reduce disaster risks, and mitigate climate change-driven impacts. On the other hand, parks being a shared place for the users, channel community participation and provide a “sense-of place” as well as improve community connections.

## 4. Discussion

Enhancing urban resilience is gaining traction as most of the global population moves to cities and risk components increase [84]. In this situation, development activities need to accommodate adaptation measures for mitigating risks and coping with new and existing challenges [85]. NbSs as adaptation and mitigation alternatives are increasingly becoming popular and justify the need for restoring urban ecology and enhancing resilience. Being one of the maximum risk-prone regions, South Asia presents an opportunity for conducting an evidence-based review of the practices and prospects of NbSs [86]. The current study explores the said opportunity by studying five cities in five countries: Barishal of Bangladesh, Phuentsholing of Bhutan, Gurugram of India, Kathmandu of Nepal, and Colombo of Sri Lanka. Each city has its distinctive urban ecosystem; two cities each with coastal and mountainous geography and one landlocked city. The risk profile of individual cities describes the specific nature of the hazard, vulnerability, coping capacity, and exposure scenario. Assessment of risks and initiatives helped the authors to present the appropriate contexts for NbR opportunities.

### 4.1. Common Disasters in Cities under Consideration

The central finding of this research is that cities under consideration suffer from different disaster events, some cities are more prone to one or two prominent disasters while others are at the risk of multiple disasters. Many global disasters have been well represented in all cases, emphasizing the fact that the South Asian region where these cities are located is among the most hazard-prone areas of the world.

#### 4.1.1. Floods, Droughts, and Rainfall Induced Landslides

By now it should be apparent that all cities have been more or less affected by disasters related to the extremes of the water cycle: floods, droughts, and rainfall-induced landslides as well as which have indirectly linked to water. Without a doubt, floods are the most common and widespread disaster that affects all five cities. It should be recognized, however, that in Phuentsholing and Colombo, floods are the most prominent disaster with frequently occurring rainfall with high intensity and of prolonged duration. In Barishal too, floods play a major role in making the city vulnerable to a variety of impacts. The extensive low-lying flood plain where the city is located makes the area highly vulnerable to frequent flooding [87]. The South-West monsoon affects all these cities with significant spatial and temporal variations in precipitation, wind speed, and temperature which could be a prominent contributory factor to increased flood damage [88]. In all cases, expansion of urban areas to accommodate rising population and unsustainable activities such as encroachment and filling of water canals, and solid waste dumping have led to increased floods. In many cases, blocked canals and poor drainage systems have resulted in increased flood duration. Colombo, in addition, experiences the effect of old and inadequate infrastructure for drainage which exacerbates flooding. Landslides frequently occur with increased rainfall and hence are very common to happen with floods. Kathmandu city experiences landslides as many slopes in the city have been altered due to human activities including construction and urban expansion. This phenomenon is also observed in Phuentsholing, which is situated in the foothills of the Himalayan Mountain region.

Although many studies acknowledge increased drought hazards in the South Asian region [89] where perennial drought prone areas are on the rise [90]. These cities in comparison do not record prominent impacts. Nevertheless, Gurugram has experienced severe and prolonged drought due to decreased precipitation.

#### 4.1.2. Threat of Multi-Hazards

On the other hand, most of the long-established and common global hazards, including hydro-meteorological hazards (wildfires, hailstorms, hurricanes, heat waves) and geo-hazards (earthquakes and landslides) continue to threaten the contemporary cityscapes of Kathmandu and Gurugram. These two cities in addition are exposed thus to multi-hazard events of floods and drought. Active volcanos have not been reported in any of these cities.

In contemporary times as well as in history Kathmandu city has been recognized as a place with multiple natural-hazards [91]. Large earthquakes have occurred sporadically, and the risks are known to increase spatiotemporally [92]. The frequency of landslides, snowstorms, hurricanes, and floods has been on the rise. Cascading effects as a result of such disasters are possible. The geographic characteristics which include closeness to Himalayan Mountains and tectonic activities make Kathmandu a “valley of disasters”. The city has faced rapid urbanization without proper planning, leading to severe environmental problems. Gurugram city, too, experiences multiple threats from a wide array of hazards ranging from floods to drought as well as from lightning hazards.

### 4.2. Lessons Highlighted: How Nbs Could Present Solutions

The present study of the five cities provides a significant amount of information that could shed light on disaster risk reduction and urban safety. For a broader view and certainly for novel approaches in this context, comparative studies are crucial to comprehend each other. This article, therefore, premised on the notion that, a comparative study facilitates to better understanding the “similarities in diversity” concerning disasters in different cities which are compared across and presents the opportunities to adopt NbSs. Multilayered context of disasters that each city experiences call to rethink disaster smart urban planning that blends with nature.

#### 4.2.1. Floods—Barishal

As a coastal city that frequently experiences extreme events, revival and restoration of its water bodies (canals and rivers) are considered necessary for enhancing NbR. However, lack of a holistic, participatory approach from the government and all other stakeholders often does not let achieve the required amount of success in restoring natural features of the city by practicing NbSs.

#### 4.2.2. Landslides—Phuentsholing

The city of Phuentsholing has introduced the “greening the city” program with strong elements to lower the susceptibility to landslides. Activities include tree planting to slope stabilization, increasing slope strength, and erosion control. These initiatives reduce landslides as well as flood risks which could otherwise be immense given the sensitivity of the area. As landslides are one of the most devastating and recurring disasters in the Himalayan region [93] adoption of NbSs, if carefully planned, may offer encouraging results.

#### 4.2.3. Droughts—Gurugram

To seek solutions to reduce the impacts of prolonged droughts, water stress, and heat waves, Gurugram has implemented new strategies with blue-green development plans. Urban green, urban forests, bioswale and green boundary, construction of retention/detention ponds, and wastewater treatment and reuse were introduced primarily to enhance water security. The city management offers “smart solutions” to link decision-makers and the community using modern communication techniques that include mobile apps and computer assisted decision support systems.

#### 4.2.4. Earthquakes—Kathmandu

Kathmandu experiences deadly earthquakes with losses and deaths on a massive scale [66]. As a popular mountainous city, Kathmandu faces rapid urbanization without proper planning, leading to severe environmental problems and loss of ecosystem services. The need for practicing NbSs through restoring green open spaces, recovering encroached river basins, reforestation of hills for stabilization, and having planned and integrated development ensuring community participation and engagement is to be addressed urgently.

Kathmandu Metropolitan City Risk Sensitive Land Use Plan presents risk-sensitive land use planning potential for mainstreaming ecosystem-based solutions. This plan recognizes increasing urban green cover, stabilizing slopes, and reforestation as remedies to prevent or control disasters and especially following disasters to “build back better”.

#### 4.2.5. Floods—Colombo

Colombo is increasingly facing the threats of floods, due to multiscale risk factors including population expansion and unsustainable development. As a solution, the wetlands of the city and suburbs were restored, connected to function as a massive “wetland unit”. As a wetland city, Colombo is enjoying the gifts of nature with additional support from newly constructed wetlands that provide multiple functions towards disaster resilience, climate change adaptations, and healthy urban life. In this new identity, the network of wetlands with connections to previously isolated wetlands, water bodies, and marshes act as state of art, strong “core” of enhancing NbR.

### 4.3. A Way Forward

The main contention in this article is that NbSs can generate new perceptions that suggest the multiple ways through which issues of similar and different nature could be handled. All five cities portray the need to achieve urban resilience through having balanced development with proper concerns for restoring, maintaining, and managing natural features and systems within the urban territory. The successful cases strengthen the concept of NbSs, highlighting the inherent affinity of human beings to become connected to natural systems for achieving NbR.

Even though this article mainly addresses the salient features of activities planned and implemented to include NbS in disaster risk reduction, some outstanding aspects are also apparent. In recent years, the role played by natural ecosystems in enhancing community well-being in multiple ways has been recognized. The cases presented here, highlight some noteworthy benefits of restoration of habitats. Firstly, cities including Gurugram and Colombo demonstrate the development of natural areas as aesthetically pleasing places for leisure, recreation, and education which could affect the psycho-social wellbeing of the communities. Secondly, deviating from the traditional disaster risk reduction paradigm of the top-down approach to new proactive responses with community participation as demonstrated in Barishal. Thirdly, in the case of Gurugram, the use of new technologies as a means of support for inclusivity and local relevance of risk communication and community resilience building.

## 5. Conclusions

The present study was carried out to investigate and report different NbSs practiced in five cities in order to reduce disaster risk and increase resilience.

Many cities in South Asia are experiencing a plethora of impacts due to natural disasters and climate change. This has resulted in increased severity of environmental and socio-economic issues. Hence, disaster risk reduction and resilience building have become key factors in ensuring the continued sustainability of cities. Against this backdrop, this paper presents some experiences in adopting NbSs to achieve urban sustainability in five cities representing five countries, i.e., Barishal (Bangladesh), Phuentsholing (Bhutan), Gurugram (India), Kathmandu (Nepal), and Colombo (Sri Lanka). Despite these cities varying in size, geography, and socio-political structures, they find common ground in terms of the catastrophic consequences of natural disasters.

In this study, the prominent disasters in each city have been compared. Simultaneously, the study also looks into the restoration, conservation and management of natural ecosystems so as to ensure resilience as a “free service of nature”. More importantly, remedial measures that are practiced by improved ecosystems and their functioning are also highlighted. The remedies presented in this study vary from restoring natural canals and wetlands to increased urban green spaces and restoration programs. This study also offers new insights into the different methodological approaches to practice NbSs, the key players related to management and community participation towards enhancing NbR.

The finding suggests that urban ecosystems, when managed properly, provide many opportunities for a wide array of benefits to the community and to the city. Related to the notion of sustainability, improved natural infrastructure could provide additional benefits towards sociological wellbeing, community participation, education and recreation. This study thus provides a wide array of new ideas, insights and tools for the policy makers and city planners to support the fostering of sustainable cities powered by NbSs.
